# Supplementary material for: miR-29b contributes to multiple types of muscle atrophy
Source: Nat Commun. 2017 May 25;8:15201. doi: 10.1038/ncomms15201 (PMC5458521; doi:10.1038/ncomms15201)
Supplement: Supplementary Information — Supplementary figures and supplementary tables. [file ncomms15201-s1.pdf]

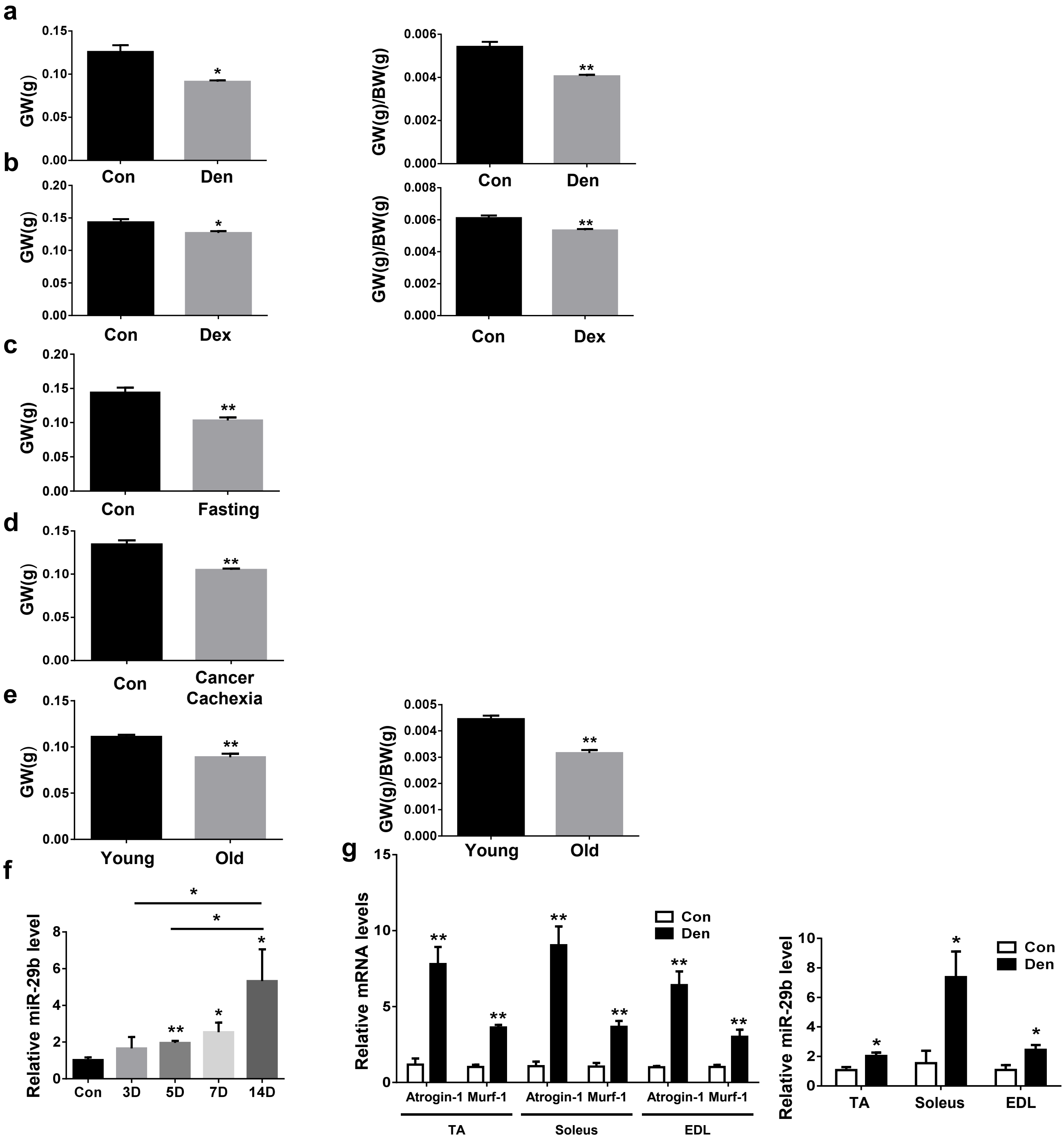

Supplementary Figure 1. Multiple types of muscle atrophy *in vivo*

(a) Gastrocnemius muscle weight (GW) and gastrocnemius muscle weight/body weight (GW/BW) ratio were reduced in denervation (Den) mice compared to control (Con) group (n=5 per group). **(b)** GW and GW/BW ratio were reduced in dexamethasone (Dex)-treated mice compared to control group (n=5 per group). **(c)** GW was reduced in fasting mice compared to control group (n=5 per group). **(d)** GW was reduced in cancer cachexia mice compared to control group (n=5 per group). **(e)** GW and GW/BW ratio were reduced in ageing (Old) mice compared to control (Young) mice (n=4 per group). **(f)** qRT-PCR analysis of miR-29b expression in gastrocnemius muscle from denervation rat at day 3, 5, 7, and 14, compared to controls (n=5 per group). **(g)** qRT-PCR analysis showed increased *Atrogin-1*, *Murf-1*, and miR-29b expressions in tibialis anterior (TA), soleus, and extensor digitorum longus (EDL) from denervation mice compared to controls (n=5 per group). Error bars, SEM. An unpaired, two-tailed Student's t test was used for comparisons between two groups (**a**-**e**, **g**). One-way ANOVA test was performed to compare multiple groups followed by Bonferroni's post hoc test (**f**). *, $P<0.05$. **, $P<0.01$.

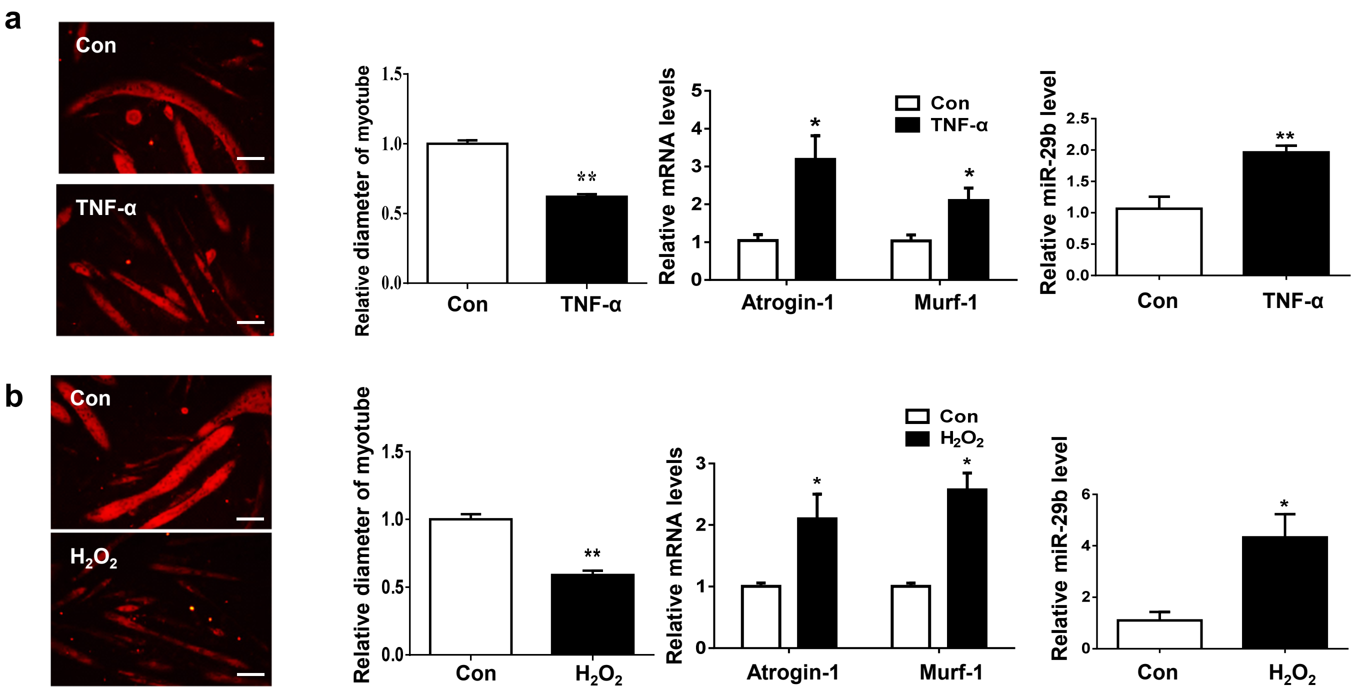

Supplementary Figure 2. miR-29b is increased in muscle atrophy cell models

(a) TNF-α (100 ng/ml) induced muscle atrophy in C2C12 myotubes as evidenced by reduced myotube diameter, accompanied with increased *Atrogin-1*, *Murf-1*, and miR-29b expression levels (n=4 per group, scale bar: 100 μm). **(b)** H_2O_2 (400 μM) induced muscle atrophy in C2C12 myotubes as evidenced by reduced myotube diameter, accompanied with increased *Atrogin-1*, *Murf-1*, and miR-29b expression levels (n=4 per group, scale bar: 100 μm). Error bars, SEM. An unpaired, two-tailed Student's t test was used for comparisons between two groups. *, $P<0.05$. **, $P<0.01$.

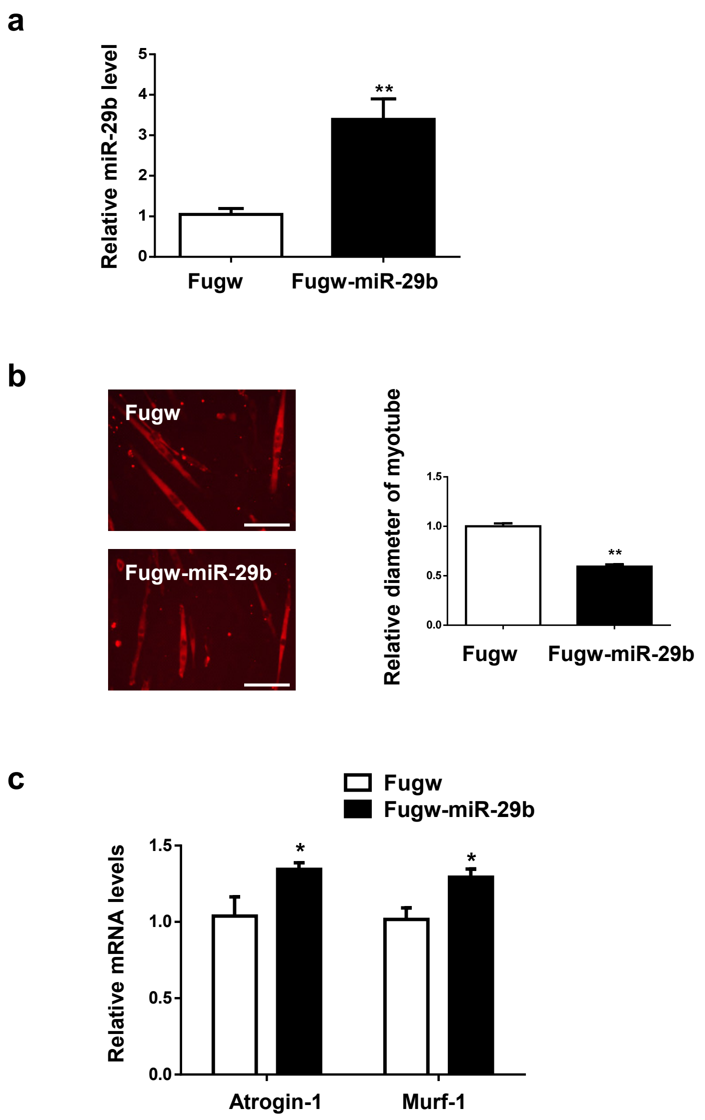

Supplementary Figure 3. miR-29b is sufficient to induce muscle atrophy *in vitro*

(a) qRT-PCR analysis showed increased miR-29b expression in C2C12 myotubes transfected with Fugw-miR-29b compared to Fugw control (n=6 per group). **(b)** Immunofluorescent staining for C2C12 myotubes showed reduced myotube diameter when transfected with Fugw-miR-29b (n=4 per group, scale bar: 100 μm). **(c)** qRT-PCR analysis showed up-regulated *Atrogin-1* and *Murf-1* expressions in C2C12 myotubes transfected with Fugw-miR-29b (n=6 per group). Error bars, SEM. An unpaired, two-tailed Student's t test was used for comparisons between two groups. *, $P<0.05$. **, $P<0.01$.

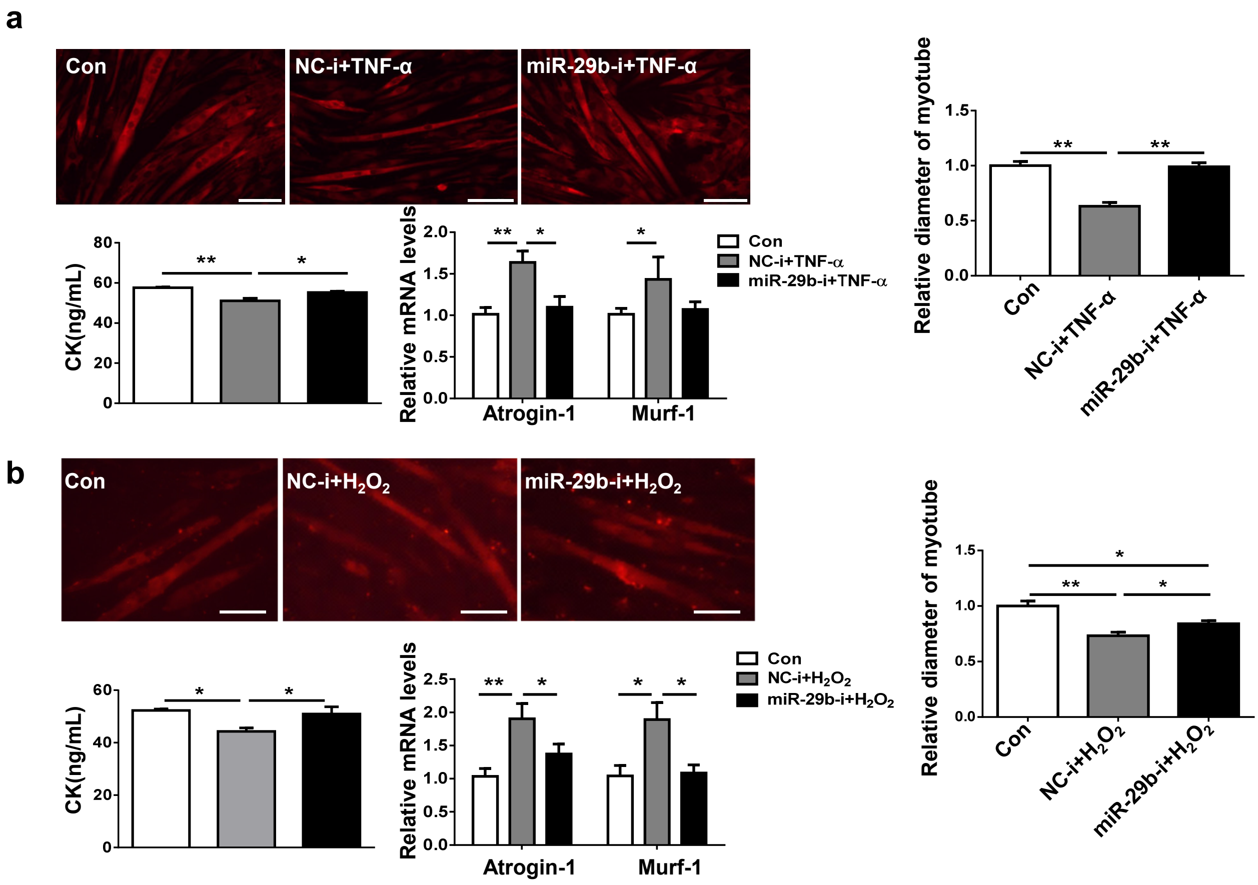

Supplementary Figure 4. miR-29b is necessary for muscle atrophy *in vitro*

(a) miR-29b inhibition attenuated TNF-α (100 ng/ml) induced muscle atrophy in C2C12 myotubes as determined by myotube diameter (n=4 per group, scale bar: 100 μm), creatine kinase (CK) activity (n=6 per group), and *Atrogin-1* and *Murf-1* expressions (n=6 per group). **(b)** miR-29b inhibition attenuated H_2O_2 (400 μM) induced muscle atrophy in C2C12 myotubes as determined by myotube diameter (n=4 per group, scale bar: 100 μm), creatine kinase (CK) activity (n=6 per group), and *Atrogin-1* and *Murf-1* expressions (n=6 per group). Error bars, SEM. One-way ANOVA test was performed to compare multiple groups followed by Bonferroni's post hoc test. *, P<0.05. **, P<0.01.

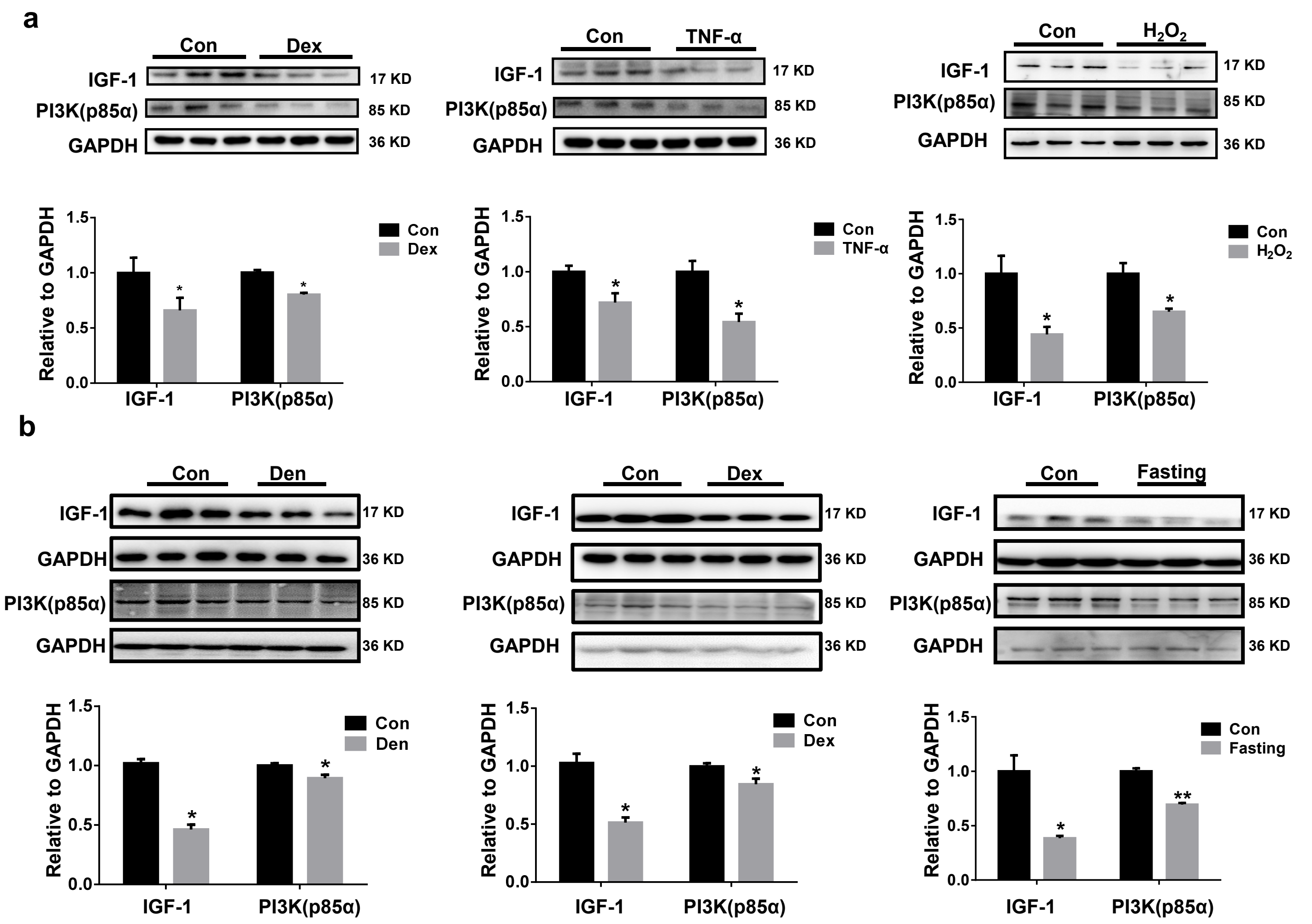

Supplementary Figure 5. IGF-1 and PI3K(p85α) are reduced in muscle atrophy

(a) Western blot showed that IGF-1 and PI3K(p85α) were down-regulated in dexamethasone (Dex)-, TNF-α-, and H_2O_2-induced muscle atrophy *in vitro* (n=3 per group). **(b)** Western blot showed that IGF-1 and PI3K(p85α) were down-regulated in denervation (Den)-, Dex-, and fasting-induced muscle atrophy *in vivo* (n=3 per group). Error bars, SEM. The presented blots are representative samples of three independent experiments. An unpaired, two-tailed Student's t test was used for comparisons between two groups. *, P<0.05. **, P<0.01.

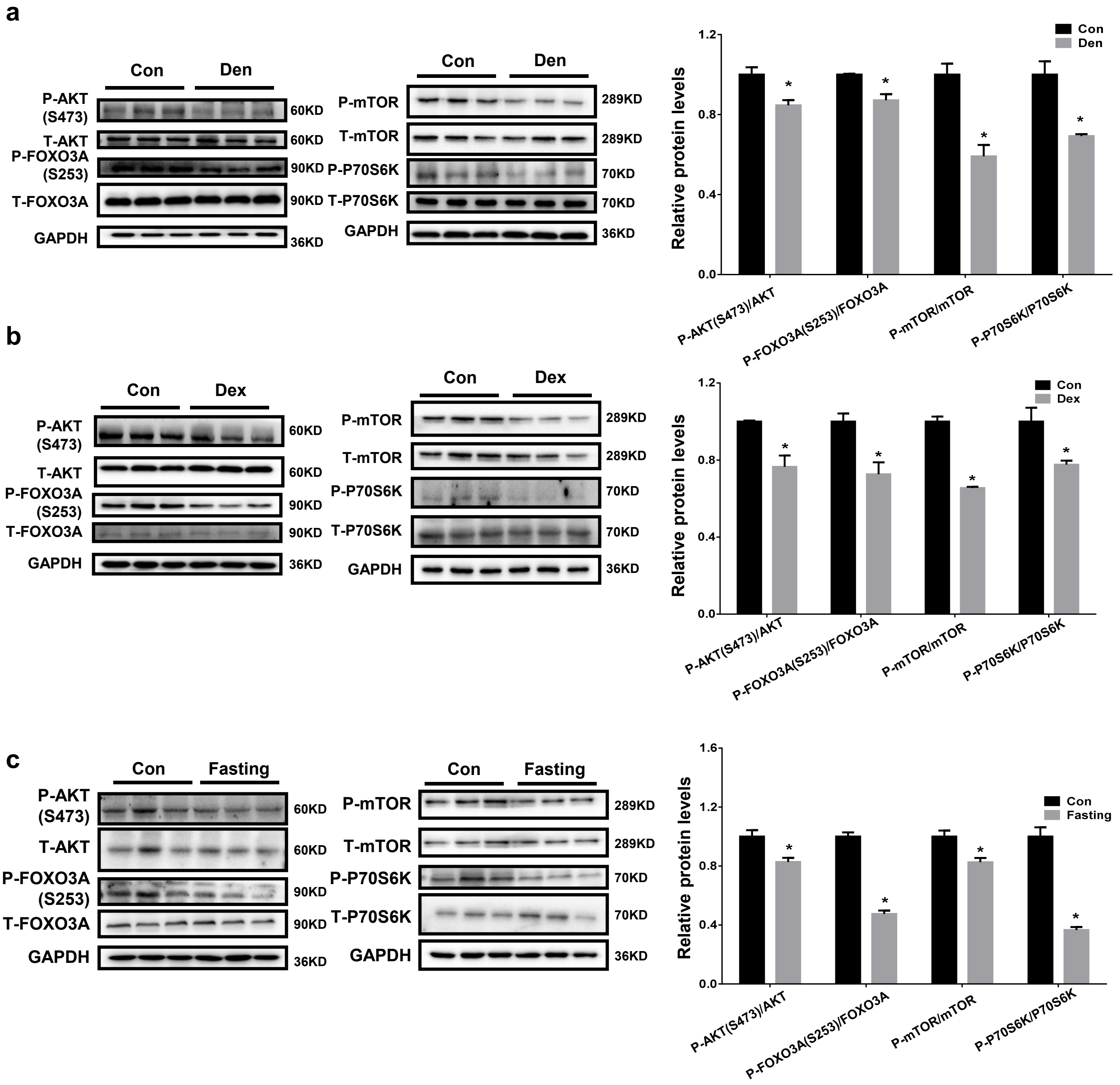

Supplementary Figure 6. IGF-1-AKT Signaling is decreased in muscle atrophy

(a) Western blot showed that the IGF-1-AKT signaling (AKT, FOXO3A, mTOR, and P70S6K phosphorylation levels) was down-regulated in denervation (Den)-induced muscle atrophy mouse model (n=3 per group). **(b)** Western blot showed that the IGF-1-AKT signaling was down-regulated in dexamethasone (Dex)-induced muscle atrophy mouse model (n=3 per group). **(c)**Western blot showed that the IGF-1-AKT signaling was down-regulated in fasting-induced muscle atrophy mouse model (n=3 per group). Error bars, SEM. An unpaired, two-tailed Student's t test was used for comparisons between two groups. *, $P<0.05$.

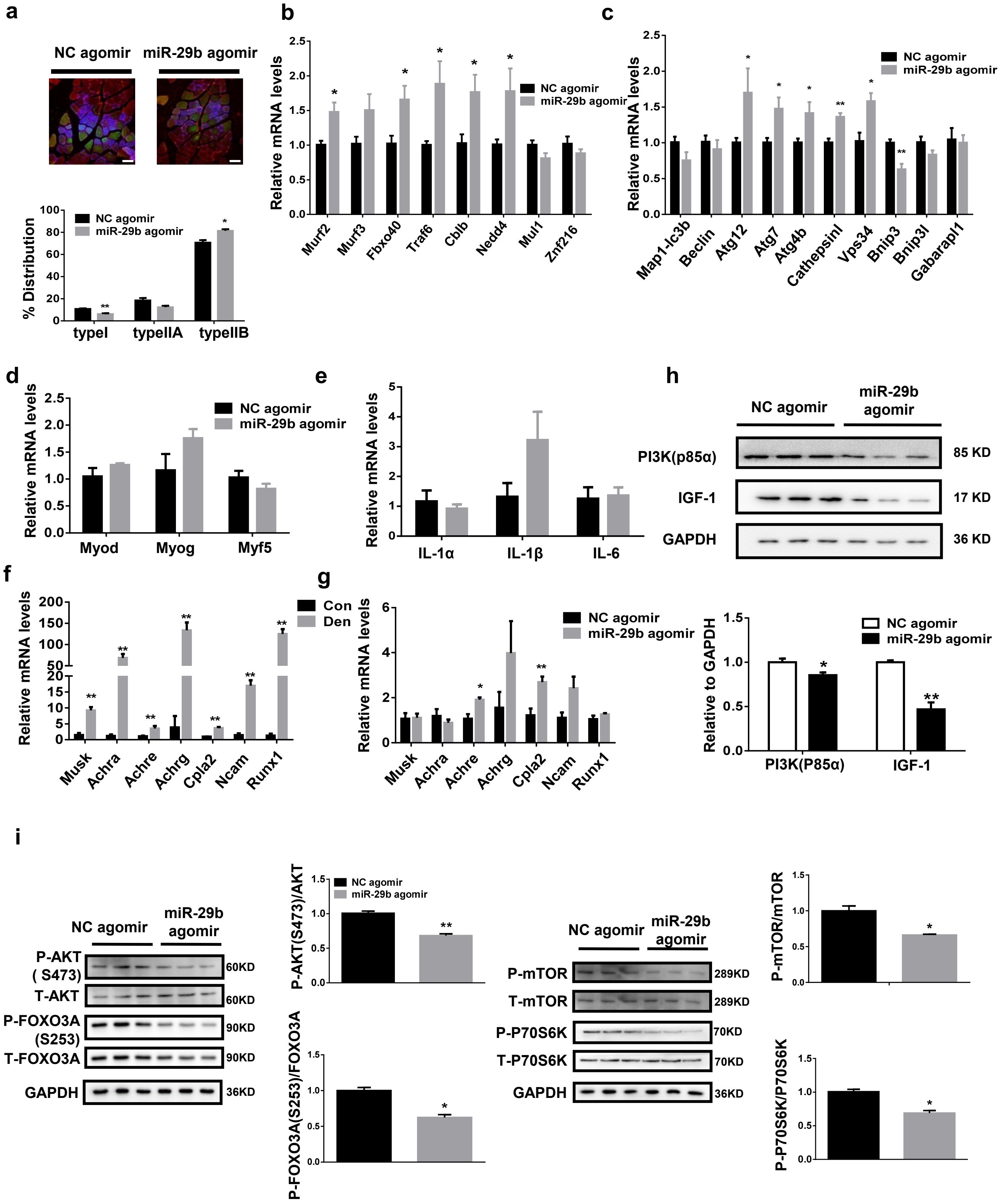

Supplementary Figure 7. miR-29b is sufficient to induce muscle atrophy *in vivo*

(a) Cross sections of gastrocnemius were stained with MHC antibodies to identify type I (blue), type IIA

(green), and type IIB (red) muscle fibers, and to quantify myofiber type distribution in NC agomir and miR-29b agomir treated mice (n=3 per group, scale bar: 50 μm). **(b-e)** qRT-PCR analysis of other ubiquitin ligases-related genes **(b)**, autophagy-related genes **(c)**, degeneration/regeneration-related genes **(d)**, and inflammation-related genes **(e)** in the gastrocnemius from mice treated with miR-29b agomir (n=5 per group)**. (f)** qRT-PCR analysis showed up-regulated transcriptional markers of denervation (*Musk*, *Achra*, *Achre*, *Achrg*, *Cpla2*, *Ncam*, and *Runx1*) in the gastrocnemius from denervated (Den) mice compared to controls (n=5 per group). **(g)** qRT-PCR analysis showed up-regulated transcriptional markers of denervation (*Achre* and *Cpla2*) in the gastrocnemius from mice treated with miR-29b agomir (n=5 per group). **(h)** Western blot showed that IGF-1 and PI3K(p85α) were down-regulated in miR-29b agomir treated mice (n=3 per group). **(i)**Western blot showed that the IGF-1-AKT signaling (AKT, FOXO3A, mTOR, and P70S6K phosphorylation levels) was down-regulated in miR-29b agomir treated mice (n=3 per group). Error bars, SEM. An unpaired, two-tailed Student's t test was used for comparisons between two groups. *, $P<0.05$. **, $P<0.01$.

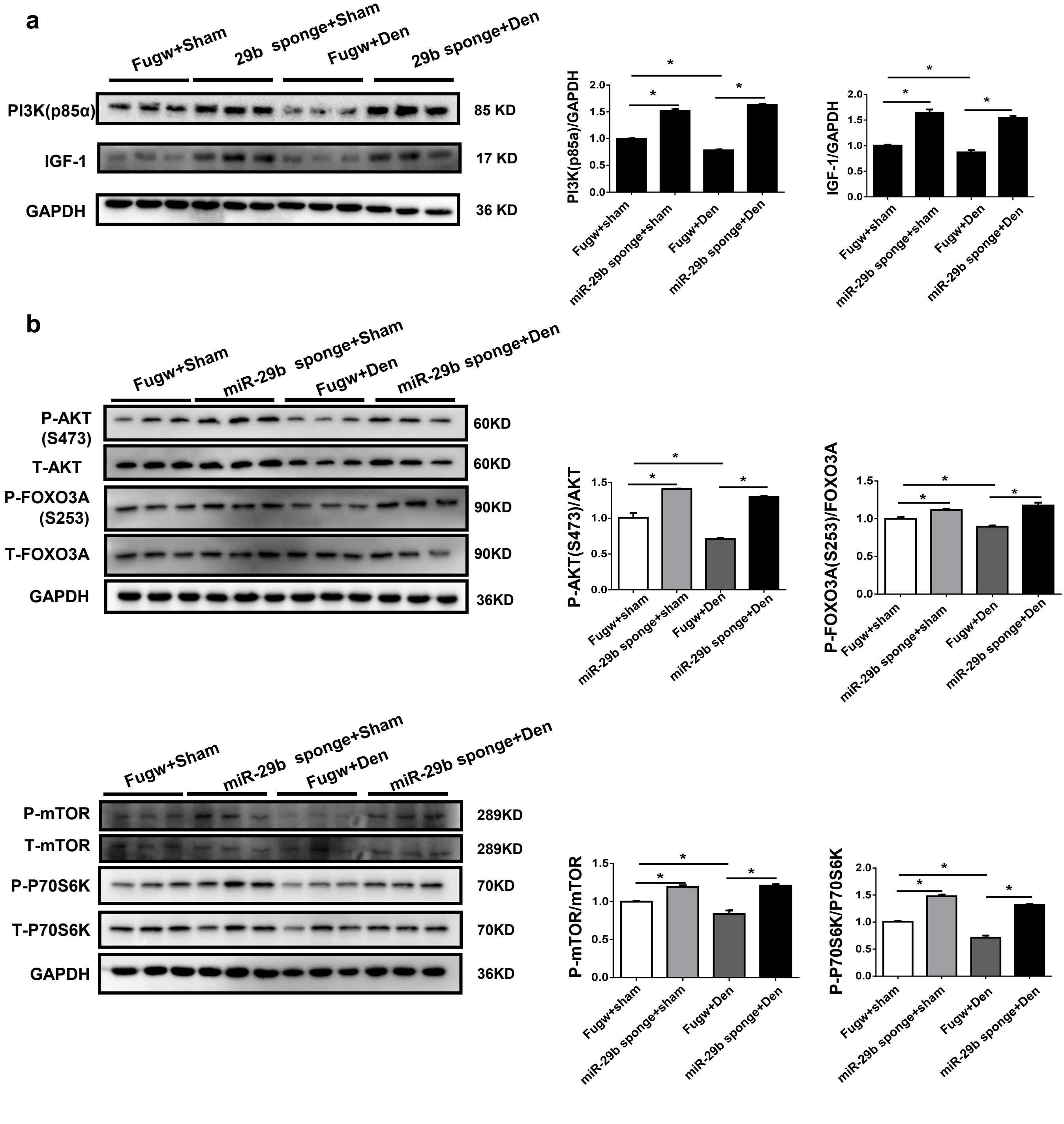

Supplementary Figure 8. miR-29b is necessary for muscle atrophy *in vivo*

(a) Western blot analysis showed that IGF-1 and PI3K(p85α) were up-regulated in the gastrocnemius from mice treated with miR-29b sponge compared to Fugw control in the presence or absence of denervation (Den) (n=3 per group). **(b)**Western blot analysis showed increased phosphorylation levels of AKT, FOXO3A, mTOR, and P70S6K in the gastrocnemius from mice treated with miR-29b sponge compared to Fugw control in the presence or absence of denervation (Den) (n=3 per group). Error bars, SEM. One-way ANOVA test was performed to compare multiple groups followed by Bonferroni's post hoc test.*, $P<0.05$.

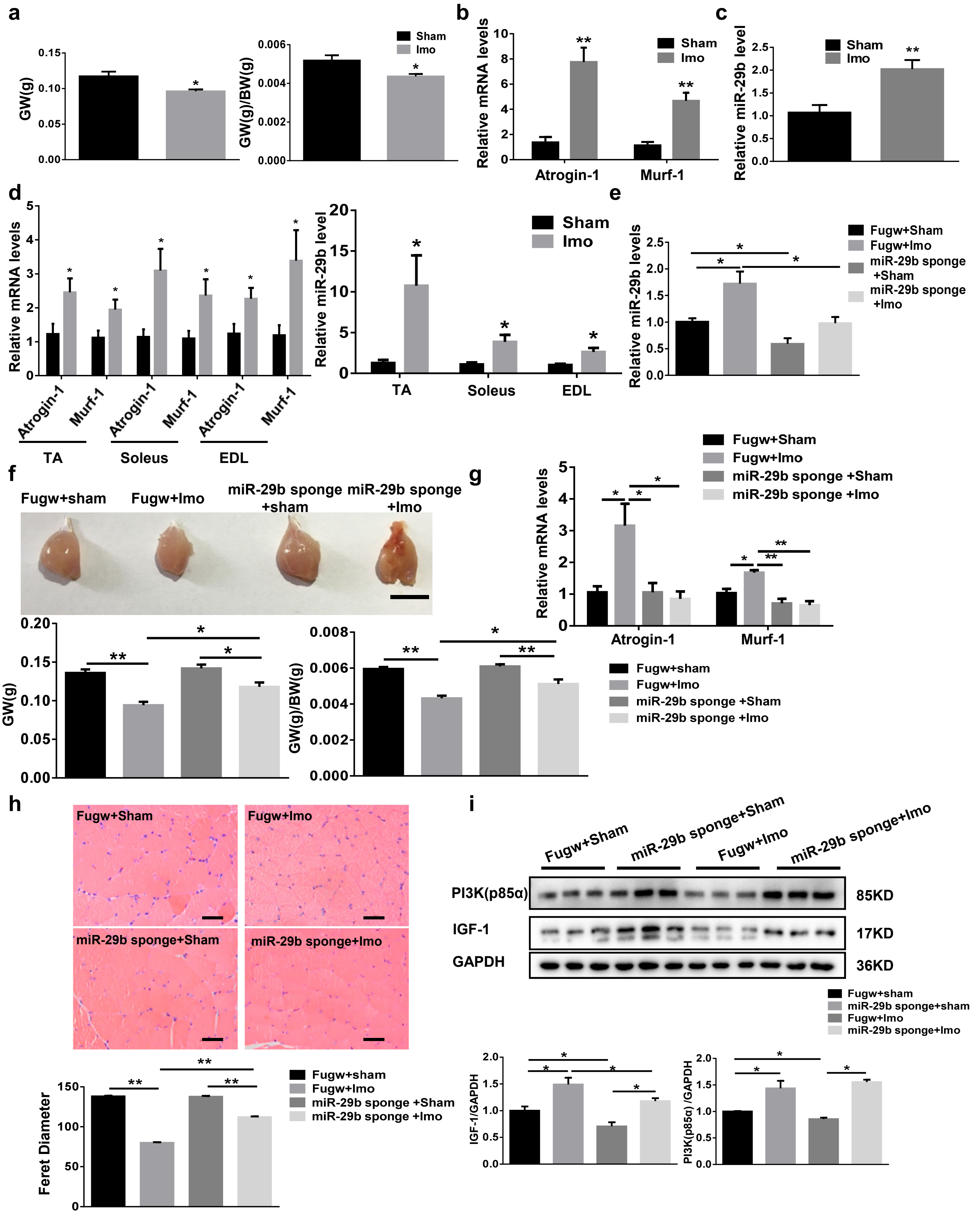

Supplementary Figure 9. miR-29b in immobilization-induced muscle atrophy

(a) Gastrocnemius muscle weight (GW) and Gastrocnemius muscle weight/Body weight (GW/BW) ratio

were reduced in immobilization mice compared to control group (n=6 per group). **(b)** qRT-PCR showed that *Atrogin-1* and *Murf-1* were up-regulated in the gastrocnemius from immobilization mice (n=6 per group). **(c)** qRT-PCR showed that miR-29b was up-regulated in the gastrocnemius from immobilization mice (n=6 per group). **(d)** qRT-PCR analysis showed increased *Atrogin-1*, *Murf-1*, and miR-29b expressions in the tibialis anterior (TA), soleus, and extensor digitorum longus (EDL) from immobilization mice compared to controls (n=6 per group). **(e)** qRT-PCR showed that miR-29b sponge significantly decreased miR-29b expression in the gastrocnemius (n=5 per group). **(f)** Gastrocnemius muscle morphology, GW, and GW/BW ratio showed that miR-29b sponge at least partly blocked immobilization-induced muscle atrophy (n=5 per group, scale bar: 1 cm). **(g)** qRT-PCR analysis showed that *Atrogin-1* and *Murf-1* were no longer up-regulated in immobilization mice treated with miR-29b sponge (n=5 per group). **(h)** Hematoxylin-eosin (HE) staining showed that the reduction in muscle fiber diameter in immobilization mice was partly attenuated by miR-29b sponge (n=5 per group, scale bar: 50 μm). **(i)** Western blot analysis showed that the down-regulation of IGF-1 and PI3K(p85α) expression levels in immobilization mice were blocked by miR-29b sponge (n=3 per group). Age and sex matched mice were used for experiments randomly. Error bars, SEM. An unpaired, two-tailed Student's t test was used for comparisons between two groups (**a-d**). One-way ANOVA test was performed to compare multiple groups followed by Bonferroni's post hoc test (**e-i**). *, $P<0.05$. **, $P<0.01$.

Supplementary Figure 10. Uncropped scans of Western blots

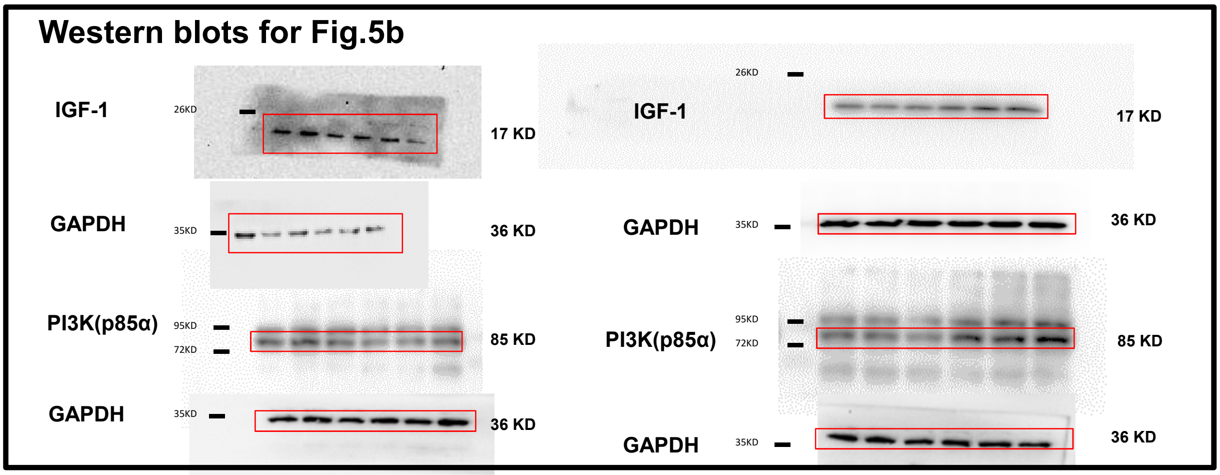

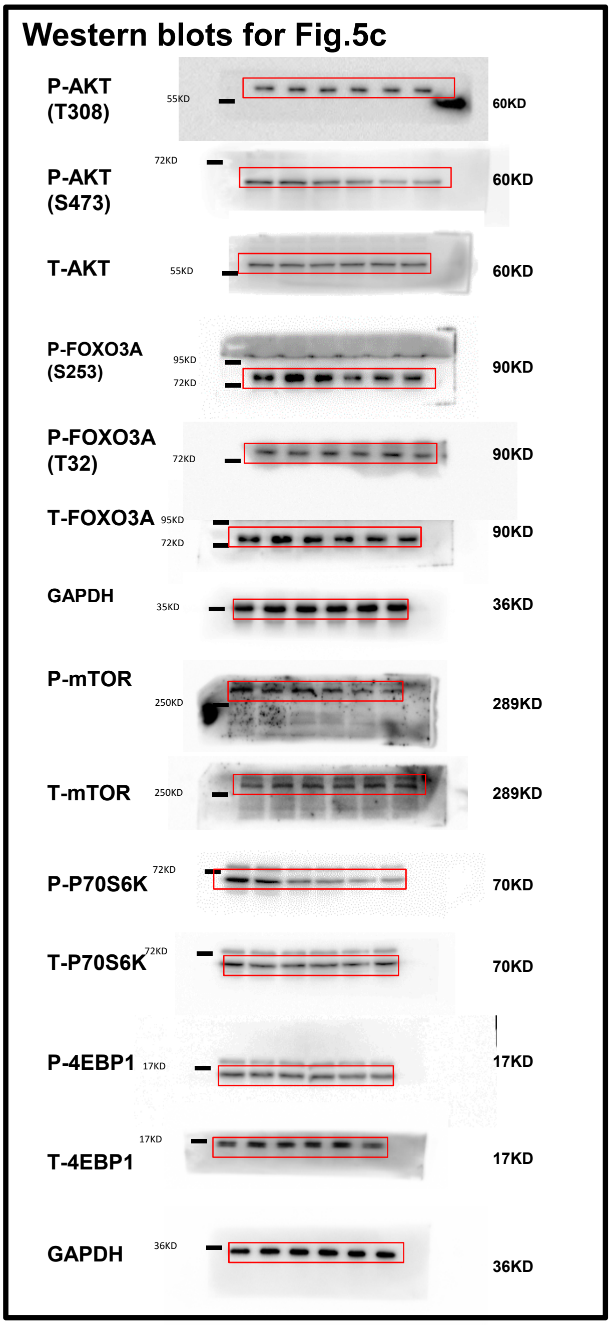

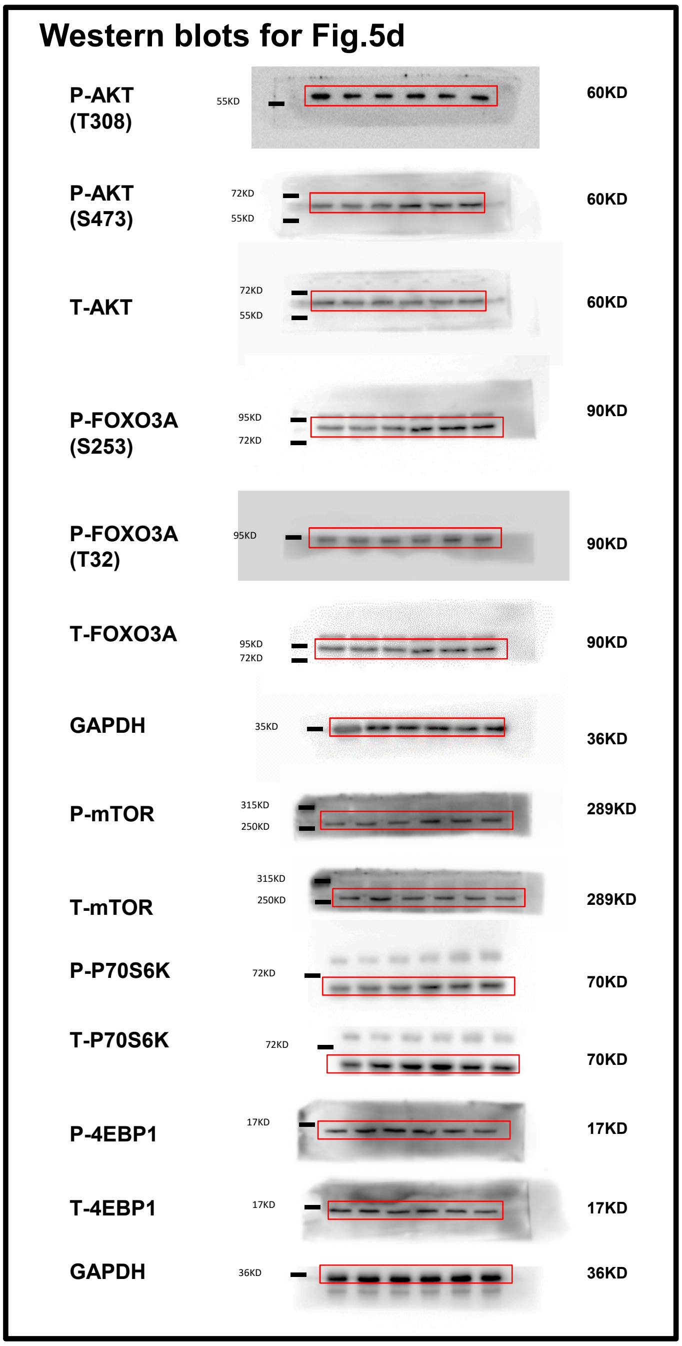

Western blots for Fig.7d

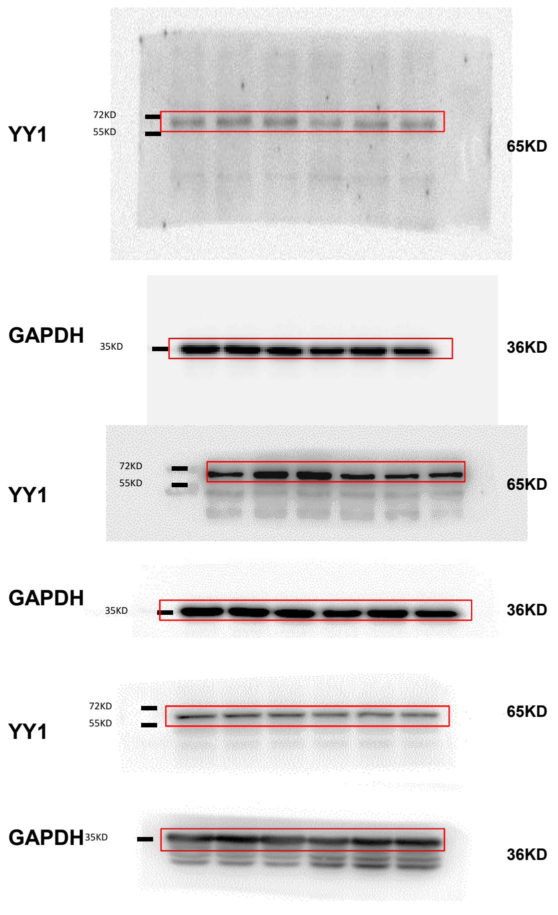

Western blots for Fig.8i

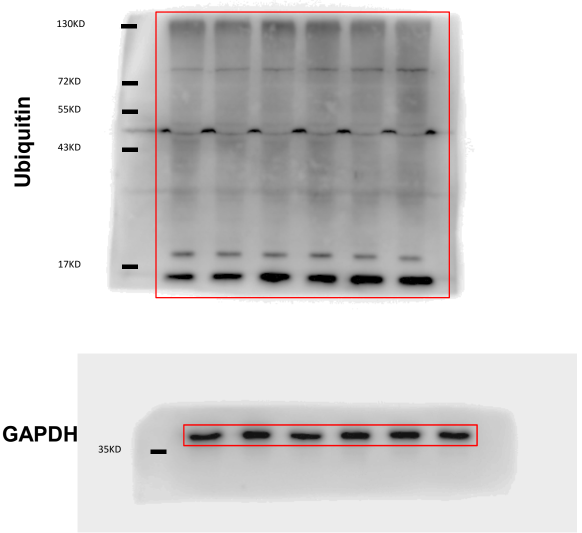

Western blots for Fig.8k

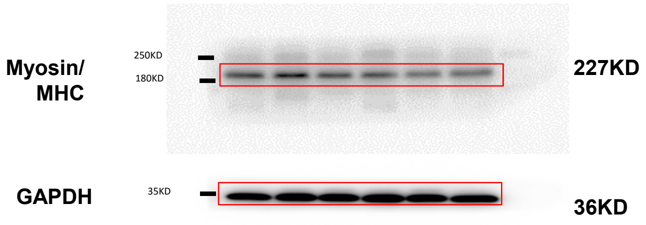

Western blots for Fig.8l

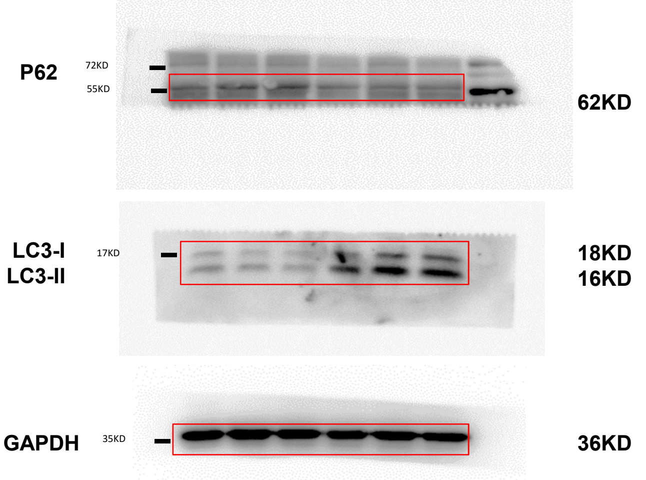

Western blots for Sup Fig.5a

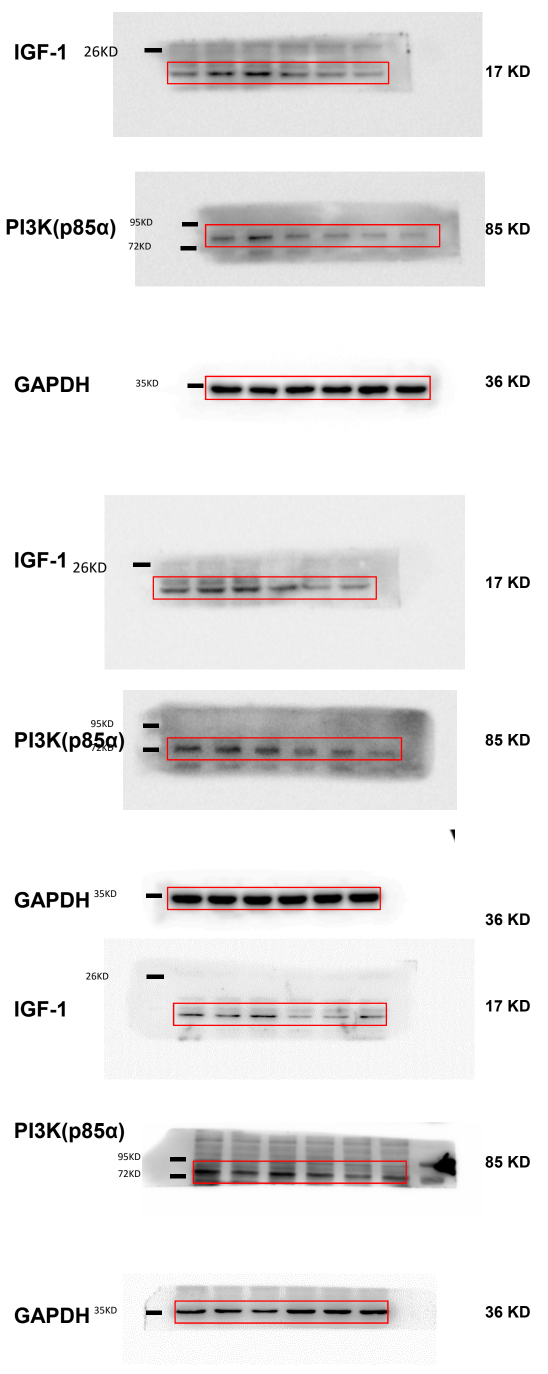

Western blots for Sup Fig.5b

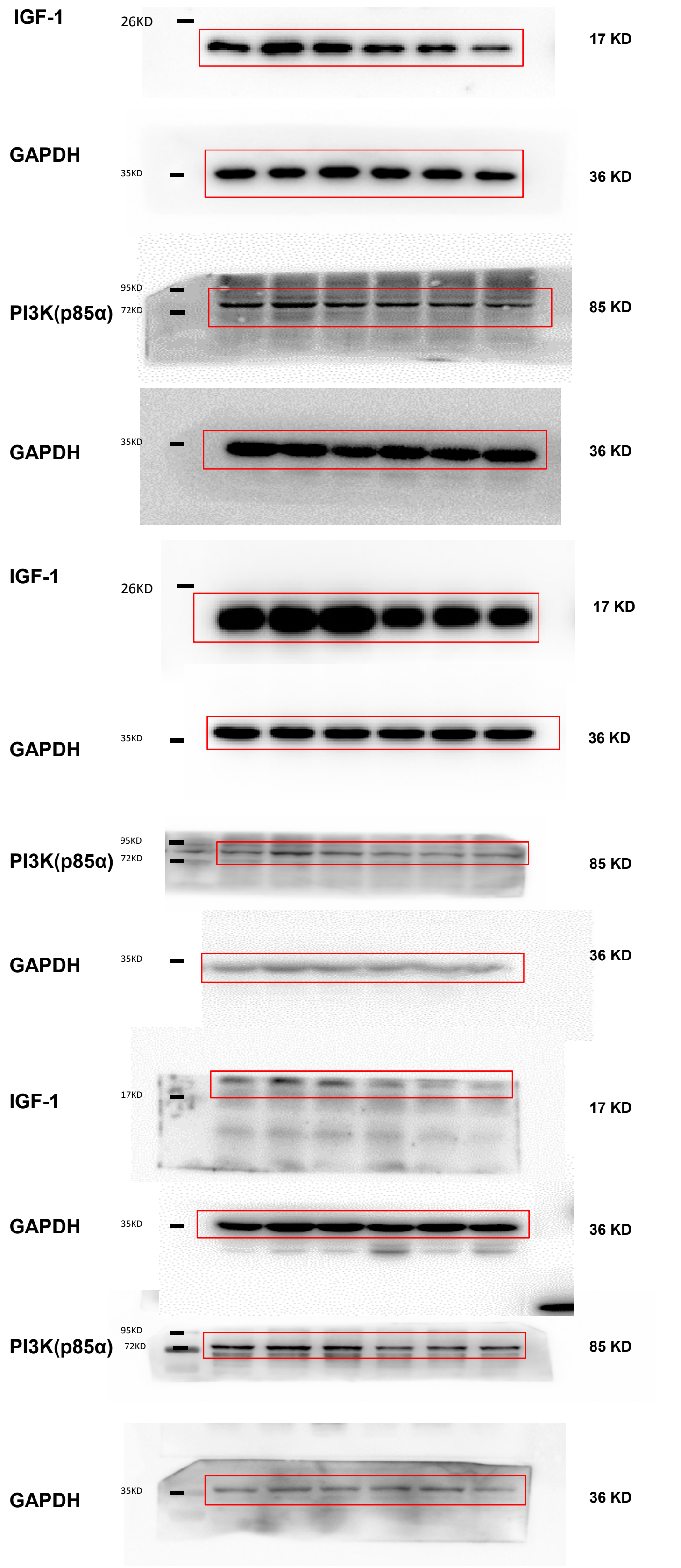

Western blots for Sup Fig.6a

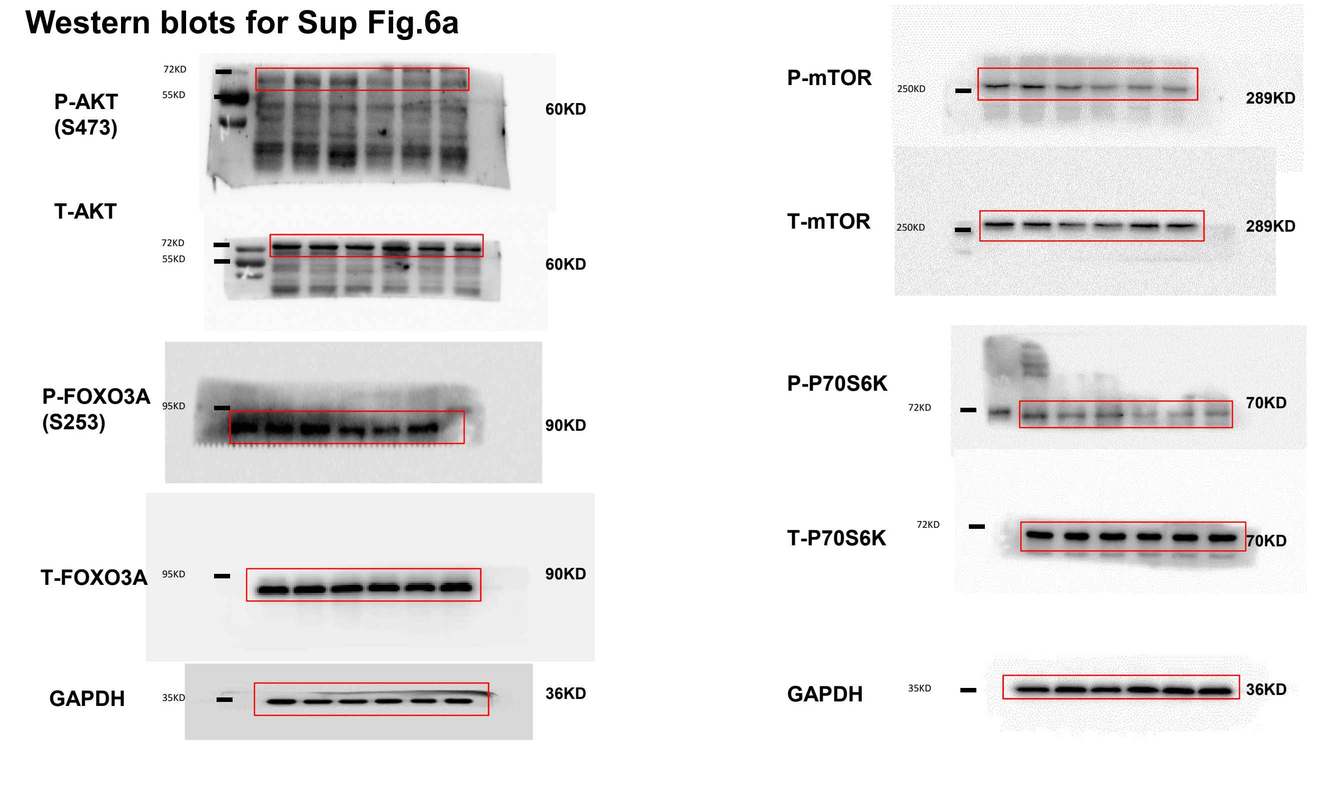

Western blots for Sup Fig.6b

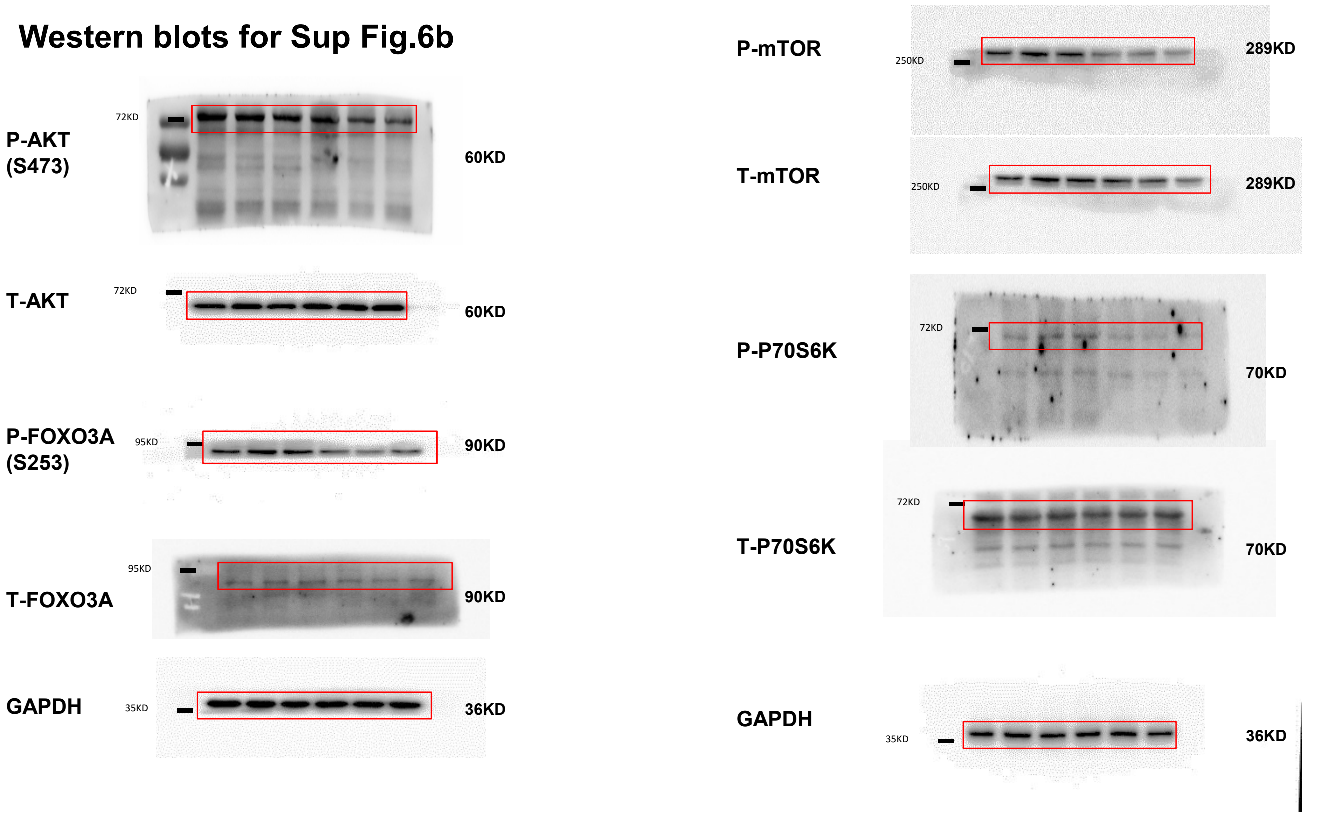

Western blots for Sup Fig.6c

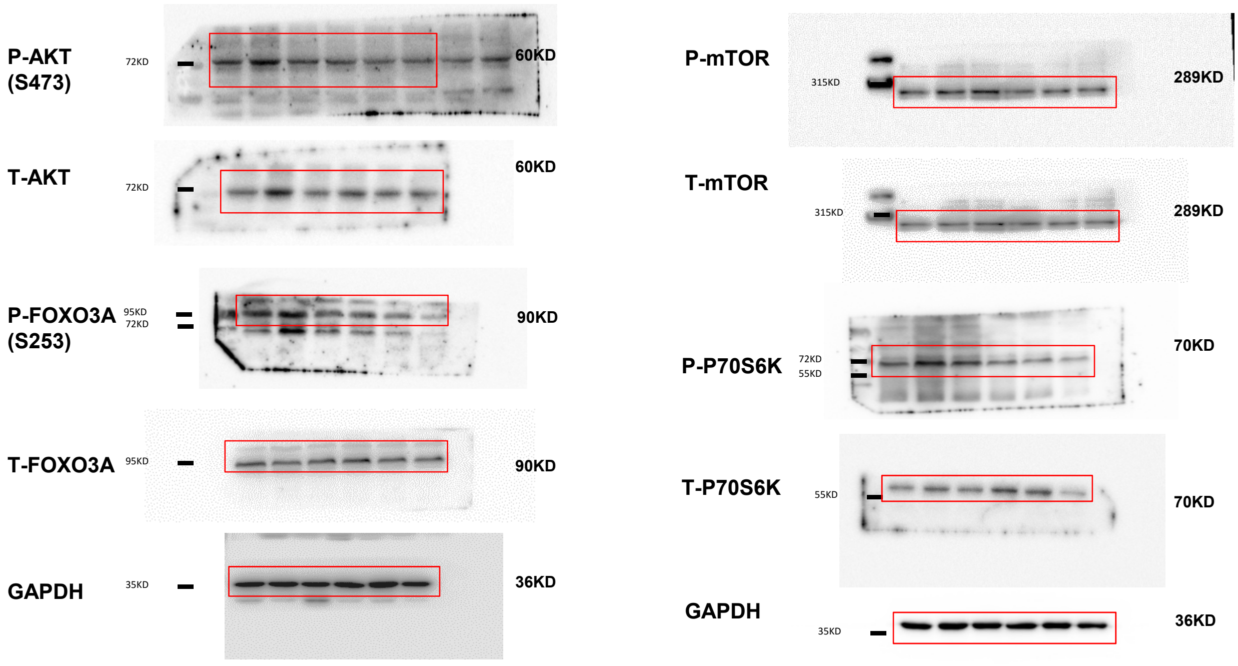

Western blots for Sup Fig.7h

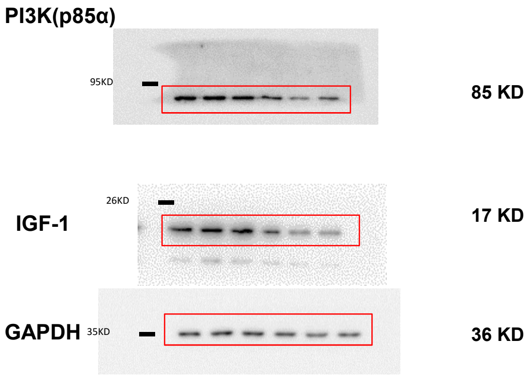

Western blots for Sup Fig.7i

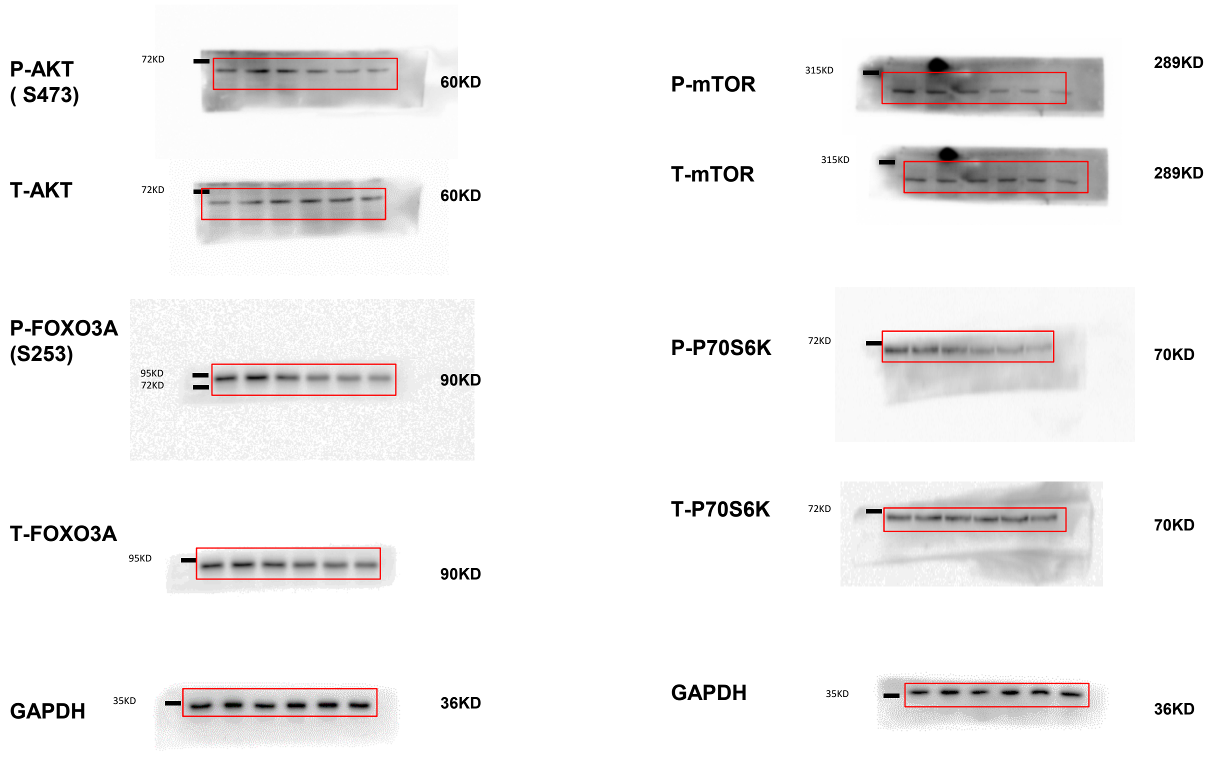

Western blots for Sup Fig.8a

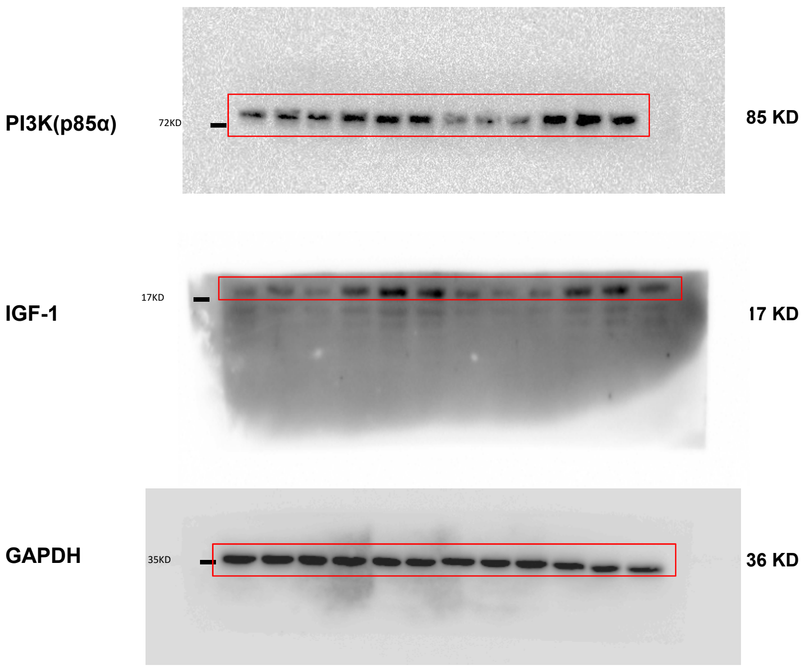

Western blots for Sup Fig.8b

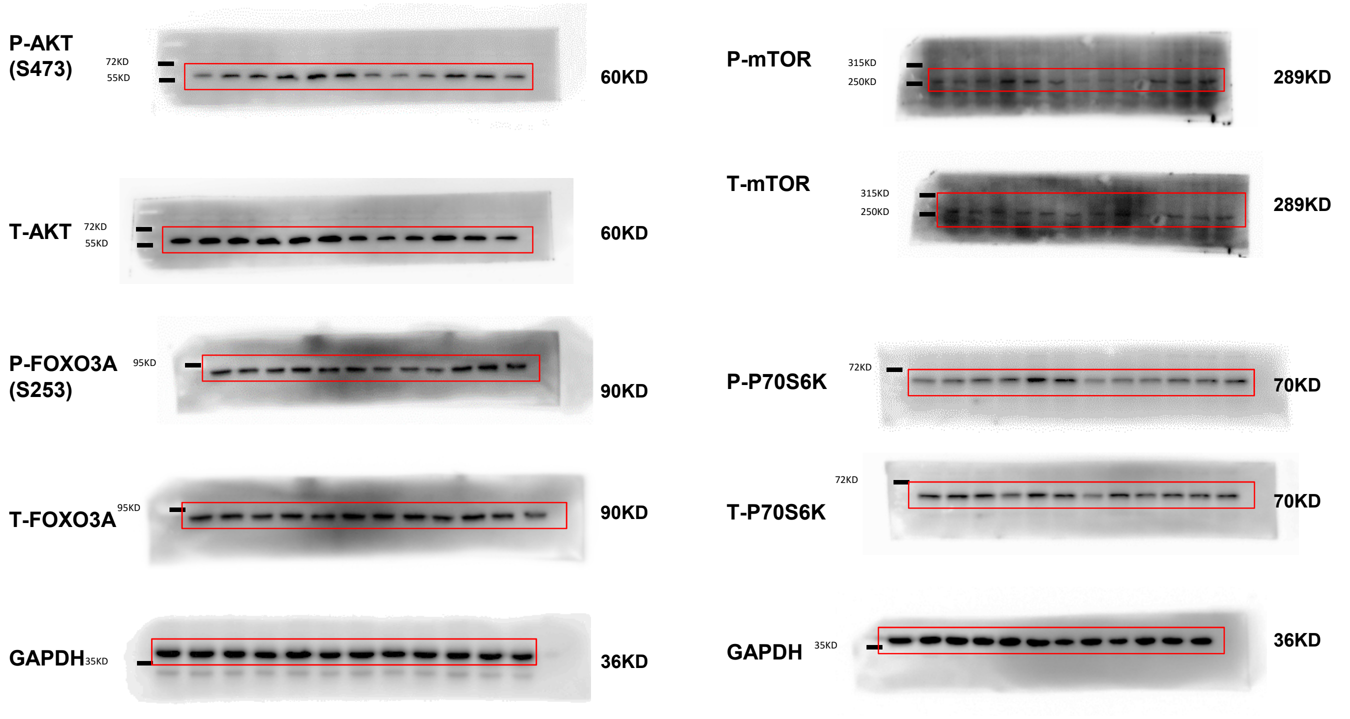

Western blots for Sup Fig.9i

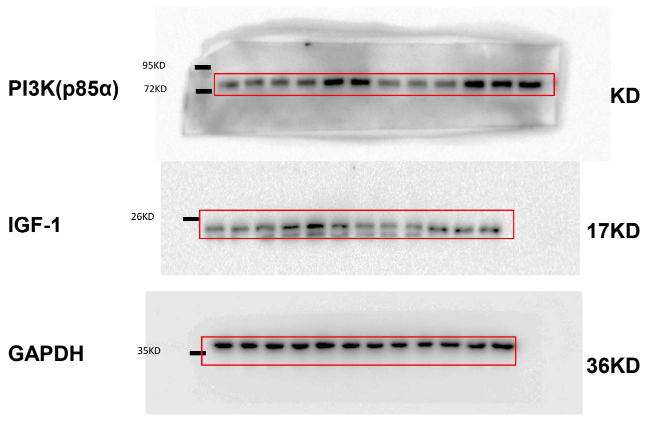

Supplementary Table 1. Aberrant expressed microRNAs in denervated muscle

MicroRNA	Regulation	Fold change	P value
miR-130b	up	6.82	0.0016
miR-21	up	6.35	0.0020
miR-29b	up	3.87	0.0039
miR-212	up	3.38	0.0143
miR-672	up	3.24	0.0344
miR-223	up	3.08	0.0081
miR-222	up	3.03	0.0001
miR-221	up	3.01	0.0002
miR-203	up	2.98	0.0135
miR-132	up	2.92	0.0060
miR-511*	up	2.77	0.0032
miR-146b	up	2.68	0.0021
miR-133a*	down	2.39	0.0086
miR-1949	up	2.38	0.0404
miR-326	up	2.21	0.0231

Supplementary Table 2. siRNA sequences used in this study

Gene	Sequence (5'-3')
mmu-Yy1	GCCCTCATAAAGGCTGCAC
	CCCTAAGCAACTGGCAGAA
mmu-IGF-1	GCCTTCCAACTCAATTATT
	GCCTCTGTGACTTCTTGAA

Supplementary Table 3. Primer sequences for qRT-PCRs used in this study

Gene	Forward primer(5'-3')	Reverse primer(5'-3')
rno-Atrogin-1	TTCCATCAGGAGAAGTGGATCT	GGCAGTCGAGAAGTCCAGTC
rno-Murf-1	CACGAAGACGAGAAAATCAACA	TGTCCTTGGAAGATGCTTTGTA
rno-18S	TCAAGAACGAAAGTCGGAGG	GGACATCTAAGGGCATCAC
mmu-MHC	GAGGGTGGCTCTCACACATTC	TTGGCCTTCGTAAGCAAACTG
mmu-Myod	CCACTCCGGGACATAGACTTG	AAAAGCGCAGGTCTGGTGAG
mmu-myog	GAGACATCCCCCTATTTCTACCA	GCTCAGTCCGCTCATAGCC
mmu-Myf5	AAGGCTCCTGTATCCCCTCAC	TGACCTTCTTCAGGCGTCTAC
mmu-IL-1α	CGAAGACTACAGTTCTGCCATT	GACGTTTCAGAGGTTCTCAGAG
mmu-IL-1β	GCAACTGTTCCTGAACTCAACT	ATCTTTTGGGGTCCGTCAACT
mmu-IL-6	TAGTCCTTCCTACCCCAATTTCC	TTGGTCCTTAGCCACTCCTTC
mmu-Yy1	CAGTGGTTGAAGAGCAGATCAT	AGGGAGTTTCTTGCCTGTCAT
mmu-Traf6	AAAGCGAGAGATTCTTTCCCTG	ACTGGGGACAATTCACTAGAGC

mmu-Nedd4	TCGGAGGACGAGGTATGGG	GGTACGGATCAGCAGTGAACA
mmu-Mul1	CTGGGCACCAGTTCGATGG	GACAGCATAAGGCACACACTT
mmu-Znf216	CCCATGCTGTGTAGTACAGGA	GCTCATTCTGCCACTATTCTGC
mmu-Fbxo40	CGTCTCCTGCCTGGTGATAAG	GTATGCTCTGACTCTTTGCACAT
mmu-Murf2	AAAGCAACTGATCTGTCCCATC	TGTGGGTAAGTACGGGTTAGAG
mmu-Murf3	GGAGAAGCAGCTCATTTGCC	CCTCCTGAAGACACCGTTGTG
mmu-Cblb	GGTCGCATTTTGGGGATTATTGA	TTTGGCACAGTCTTACCACTTT
mmu-Atg7	GTTCGCCCCCTTTAATAGTGC	TGAACTCCAACGTCAAGCGG
mmu-Map1-lc3	CACTGCTCTGTCTTGTGTAGGTTG	TCGTTGTGCCTTTATTAGTGCATC
mmu-Cathepsinl	GTGGACTGTTCTCACGCTCAAG	TCCGTCCTTCGCTTCATAGG
mmu-Gabarapl1	CATCGTGGAGAAGGCTCCTA	ATACAGCTGGCCCATGGTAG
mmu-Bnip3	TTCCACTAGCACCTTCTGATGA	GAACACCGCATTTACAGAACAA
mmu-Bnip3l	TTGGGGCATTTTACTAACCTTG	TGCAGGTGACTGGTGGTACTAA
mmu-Atg12	TCCGTGCCATCACATACACA	TAAGACTGCTGTGGGGCTGA

mmu-Vps34	TGTCAGATGAGGAGGCTGTG	CCAGGCACGACGTAACTTCT
mmu-Beclin	TGAATGAGGATGACAGTGAGCA	CACCTGGTTCTCCACACTCTTG
mmu-Atg4b	ATTGCTGTGGGGTTTTTCTG	AACCCCAGGATTTTCAGAGG
mmu-TBP	TGTGAATACTGGTGCTGAG	GGCATGAGACAAGACCTATA
mmu-Musk	TTCAGCGGGACTGAGAAACT	TGTCTTCCACGCTCAGAATG
mmu-Achra	TCCCTTCGATGAGCAGAACT	GGGCAGCAGGAGTAGAACAC
mmu-Achre	GTGTCTGGATTGGCATTGACT	ACACCTGCAAAATCGTCCTTG
mmu-Achrg	GACCAACCTCATCTCCCTGA	GAGAGCCACCTCGAAGACAC
mmu-Cpla2	GACAGCTCCGACAGTGATGA	CGTCCTTCTCGGGTATTGAA
mmu-Ncam	AAGGGGAAGGCACTGAATTT	TCTCCTGCCACTTGACACAG
mmu-Runx1	AGCCTGGCAGTGTCAGAAGT	TGGCATCTCTCATGAAGCAC
mmu-Myh1	GCGAATCGAGGCTCAGAACAA	GTAGTTCCGCCTTCGGTCTTG
mmu-Myh2	AAGTGACTGTGAAAACAGAAGCA	GCAGCCATTTGTAAGGGTTGAC
mmu-Myh4	CTTTGCTTACGTCAGTCAAGGT	AGCGCCTGTGAGCTTGTAAA

mmu-Myh7	ACTGTCAACACTAAGAGGGTCA	TTGGATGATTTGATCTTCCAGGG
muu-Atrogin-1	CAGCTTCGTGAGCGACCTC	GGCAGTCGAGAAGTCCAGTC
Mmu-IGF-1	GTGGGGGCTCGTGTTTCTC	GATCACCGTGCAGTTTTCCA
mmu-Murf-1	GTGTGAGGTGCCTACTTGCTC	GCTCAGTCTTCTGTCCTTGGA
mmu-18S	TCAAGAACGAAAGTCGGAGG	GGACATCTAAGGGCATCAC
